# Klebsiella Discitis in a 15-Year-Old Male Diagnosed With Plasma Microbial Cell-Free DNA Next-Generation Sequencing Test: A Case Report

**DOI:** 10.7759/cureus.21237

**Published:** 2022-01-14

**Authors:** Farida Karim, Marmik Patel, Lori L Barr, Paola J Maurta-Neumann, Florentina Litra

**Affiliations:** 1 Pediatrics, Sacred Heart Hospital, Pensacola, USA; 2 Pediatric Radiology, Radiology Associates of Florida-Panhandle Division, Radiology Partners, Pensacola, USA; 3 Pediatric Infectious Diseases, Sacred Heart Hospital, Pensacola, USA; 4 Pediatrics, University of Florida, Pensacola, USA; 5 Pediatrics, Ascension Sacred Heart, Pensacola, USA

**Keywords:** klebsiella aerogenes, neurosurgery, epidural abscess, spinal infections, pediatric discitis

## Abstract

Spinal discitis (SD) is a rare condition, particularly in the pediatric population. The course of SD may be acute or chronic, and the non-specificity of symptoms leads to great delays in diagnosis. The most commonly isolated causative organism is *Staphylococcus aureus* whereas gram-negative infections are hardly ever reported in the literature. Comorbidities that increase the risk of bacteremia such as diabetes, chronic kidney disease, HIV, and cancer are major risk factors for SD. Hereby, we present an atypical case of SD in a previously healthy 15-year-old male with an unusual organism, *Klebsiella aerogenes*, diagnosed by plasma microbial cell-free DNA with negative blood cultures. The clinical course was complicated by antibiotic resistance and subsequent development of a ventral epidural abscess requiring readmission followed by surgical drainage of the abscess with a prolonged course of antibiotics.

## Introduction

Pediatric spinal infections are very rare. However, they are a significant cause of morbidity among the pediatric population. These infections encompass a broad range of conditions, from discitis to osteomyelitis and spinal epidural and intramedullary abscesses [1]. Discitis is the infectious or inflammatory disease of the intervertebral disc space, which is usually self-limiting in children [2]. Diagnosis is often delayed, especially in young children, as the initial symptoms may mimic other processes such as septic arthritis, appendicitis, or meningitis [3]. The pathological mechanism of discitis is still unclear; however, trauma, infection, increased vascularity of the vertebra in the young, and inflammation have been implicated as possible etiologies [3]. Bacterial infection is undoubtedly responsible for most cases [4]. Reported risk factors include congenital heart disease, immune disorders, long-term use of vascular access devices, spinal cord tumors, and dermal sinuses [1]. MRI reduces the diagnostic delay and may help in avoiding the requirement for a biopsy since laboratory tests and cultures of blood and disc tissue are usually found to be negative, especially in the early phases of the disease [5]. General management includes rest and immobilization. Early identification and appropriate antimicrobial administration are paramount for improved prognosis, clinical outcomes, and preventing complications [1].

## Case presentation

**Figure 1:**
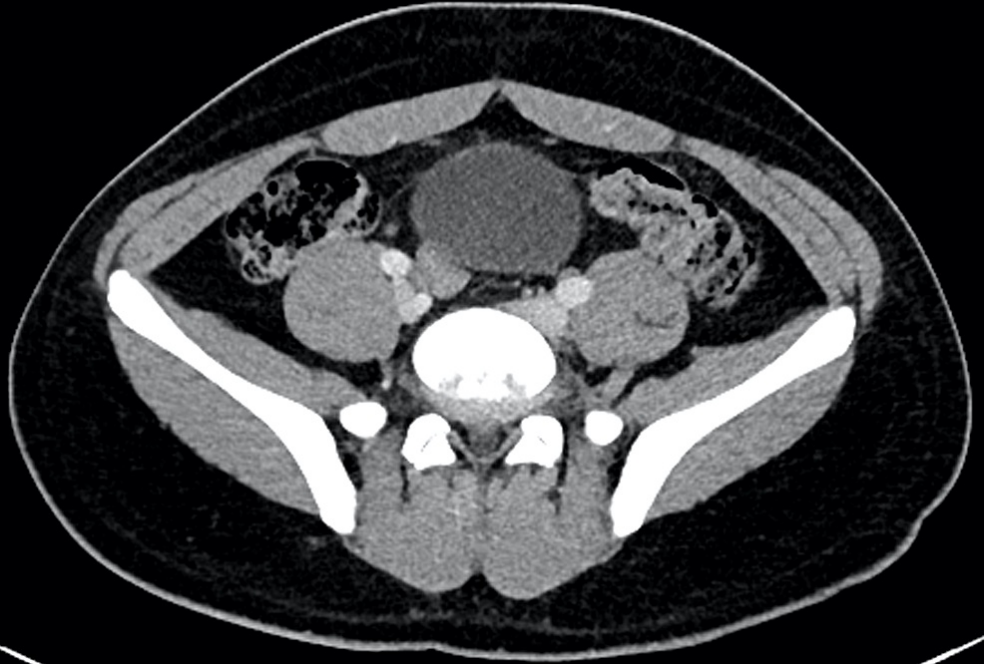
Contrast-enhanced CT scan of the abdomen/pelvis. Axial image at L5-S1 also shows the enhancement without evidence of paraspinous mass, fluid, or abnormal enhancement.

**Figure 2:**
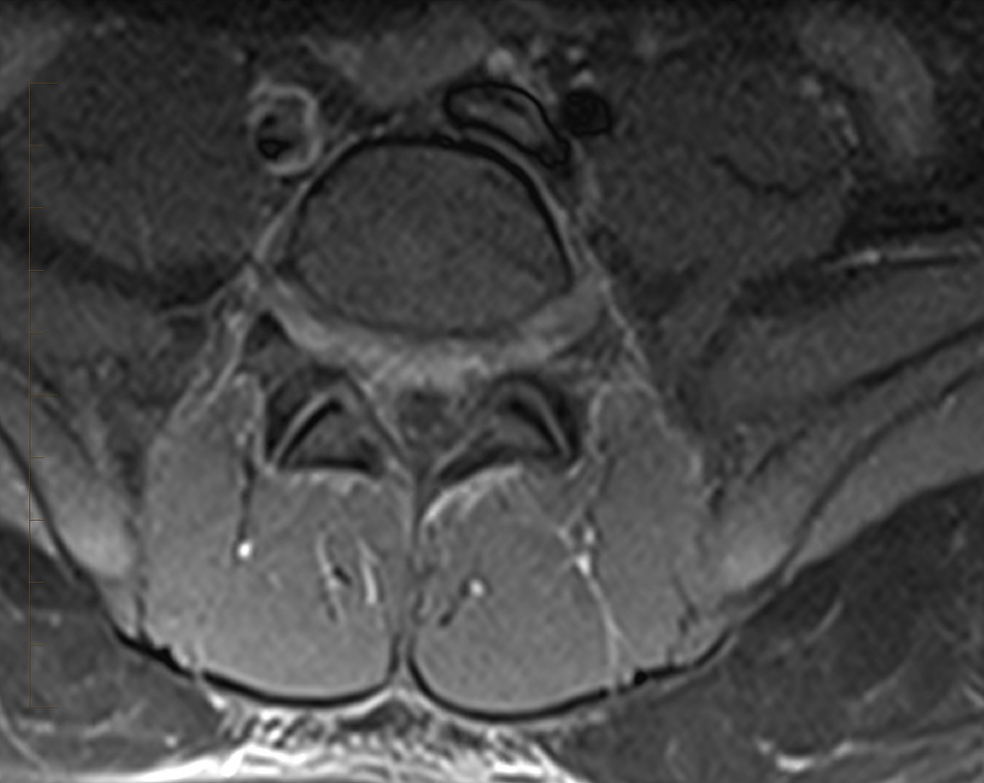
T1 fat-saturated contrast-enhanced MRI of the lumbar spine. Axial image at L5-S1 level demonstrates mild enhancement of the anterior aspect of the thecal sac.

A 15-year-old previously healthy Hispanic male presented with a two-day history of fever and lower back and abdominal pain. He was transferred from an outside hospital where an MRI lumbar spine was suggestive of lumbar spine discitis. On arrival at our hospital, he was hemodynamically stable with no acute distress. He had lower back tenderness with exacerbation of pain on even slight movement; therefore, he was unable to walk. He did not have any focal neurological deficits including sensory or motor deficits. The lab workup showed mild leukocytosis with neutrophilia, elevated inflammatory markers such as C-reactive protein (CRP) and erythrocyte sedimentation rate (ESR), and an unremarkable urinalysis. A CT scan of the abdomen and pelvis was performed to better evaluate the retroperitoneal spaces, which demonstrated early changes of discitis (Figure 1). Subsequently, neurosurgical evaluation was done and medical management with antibiotics was recommended. Therefore, the patient was started on ceftriaxone, vancomycin, and metronidazole on admission. His blood culture from admission remained negative. Four days after initiation of broad-spectrum parenteral antimicrobial therapy, a follow-up MRI of the spine was repeated to assess the response to therapy. It showed no evidence of osteomyelitis or epidural collection and mild disc degeneration at L5-S1 with small central disc protrusion, with mild edema/enhancement of the L4-L5 and L5-S1 interspinous ligaments, was noted (Figure 2).

Routine infectious and immune workups including blood cultures, QuantiFERON gold, and *Brucella* serology returned negative. Given the unremarkable history and absence of typical risk factors, a plasma microbial cell-free DNA next-generation sequencing test was performed in an attempt to identify the causative organism.

The patient was treated for infectious discitis with ceftriaxone, vancomycin, and metronidazole, with the initial improvement of symptoms. He was discharged on a prolonged course of IV ceftriaxone 50 mg/kg twice per day (BID) and orally (PO) linezolid 600 mg BID (at least a four-week course with the decision on the final duration of therapy contingent on outpatient follow-up with infectious disease). He was also discharged on a PO metronidazole 10 mg/kg three times a day (TID) to complete a full 14-day course.

**Figure 3:**
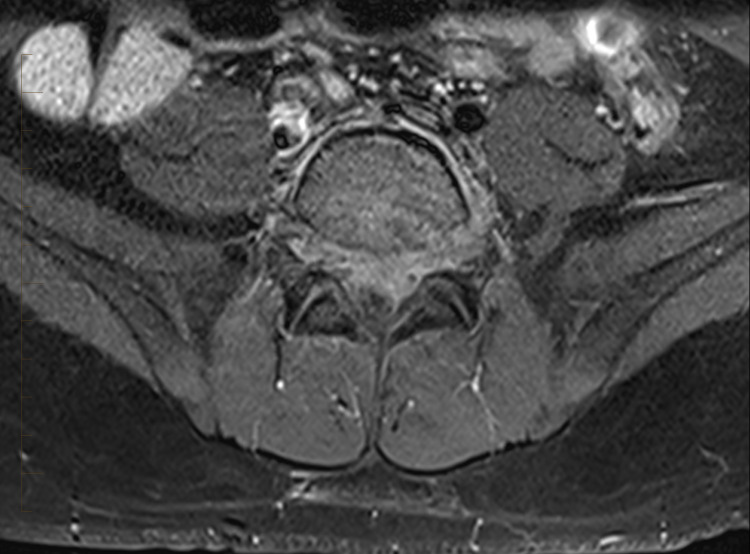
T1 fat-saturated contrast-enhanced MRI of the lumbar spine. Axial image at L5-S1 level demonstrates worse enhancement of the anterior aspect of the thecal sac and extension of enhancement along the left side of the vertebral body. The nerve roots are displaced posteriorly by epidural phlegmon.

Ten days later after his discharge, he was readmitted with worsening back pain, bilateral testicular pain, and an episode of urinary incontinence. At that time, his neurologic examination was unremarkable without any motor or sensory deficits. However, his presentation with radicular component was concerning for either worsening disc protrusion compressing spinal nerves or development of an epidural abscess. An MRI of the spine showed L5-S1 discitis with an interval increase in the size of enhancing epidural tissue extending from the top of L5 to mid S2 level. This was suspicious for an epidural phlegmon with the extension of inflammation into the left paravertebral region and new mild bone marrow edema worrisome for osteomyelitis (Figure 3).

At this time, the plasma microbial cell-free DNA next-generation sequencing test, which was sent during the prior admission, identified *Klebsiella aerogenes*. Resistance to ceftriaxone was suspected, therefore, his current antibiotic coverage was changed from ceftriaxone and linezolid to cefepime and linezolid. The patient was then taken for surgical exploration to rule out the formation of a spinal epidural abscess. Tissue and fluid cultures obtained during the surgery grew *K. aerogenes*, which showed multiple drug resistance but susceptibility to cefepime.

After initial post-operative improvement, the patient's fever and back pain radiating to the bilateral testicular area returned within 12 hours. Cefepime was changed to meropenem due to concerns of the extended-spectrum beta-lactamase (ESBL) organism.

**Figure 4:**
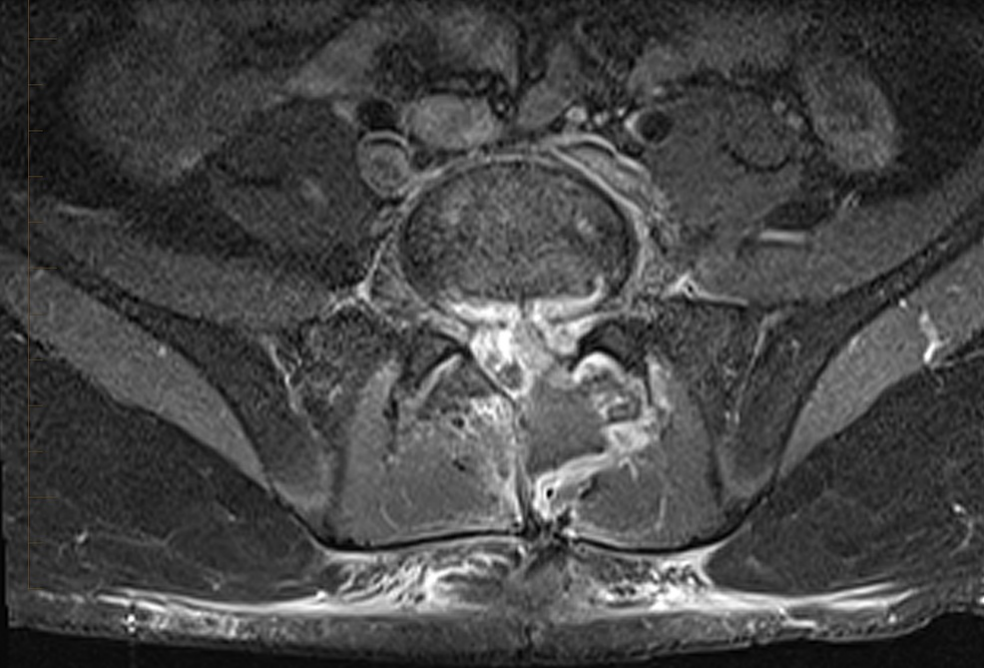
MRI lumbar spine T1 fat-saturated with IV contrast. Axial post-contrast image shows bright epidural enhancement surrounding a low signal intensity fluid collection that extends into the left paraspinous muscles and the posterior subcutaneous tissues.

A repeat MRI of the spine, six days post-operatively, showed residual ventral epidural phlegmon at L5-S1 and a ventral epidural fluid collection anterior to the phlegmon, suggestive of seroma or abscess (Figure 4). Due to persistent fever and evidence of disease progression per imaging, our patient underwent CT-guided drainage of the epidural abscess and drain placement by interventional radiology 12 days post-operatively.

Spinal fluid cultures obtained during the drainage also grew *K. aerogenes*. Following decreased drainage and improvement of the symptoms, a follow-up MRI, 11 days post-drain placement, showed improving ventral epidural phlegmon at L5-S2 with interval resolution of collection anterior to this phlegmon. The drain was, therefore, removed, and the patient was discharged on long-term meropenem therapy with close clinical surveillance by the infectious diseases specialist.

**Figure 5:**
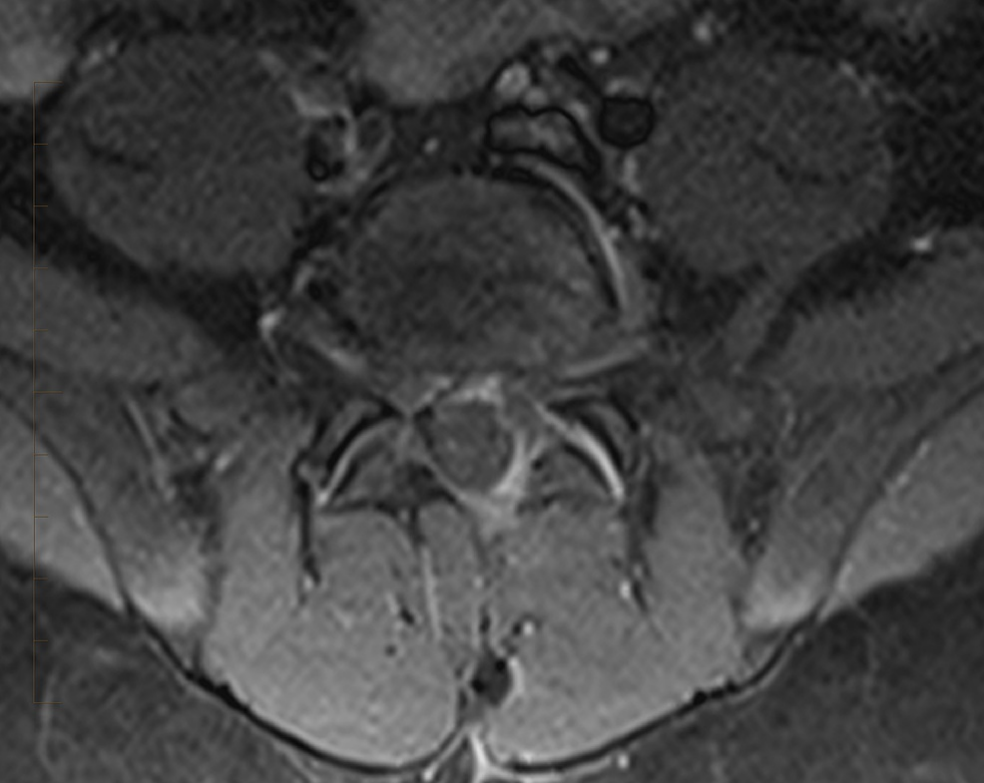
T1 fat-saturated contrast-enhanced MRI of the lumbar spine. Axial view at L5-S1 demonstrates no abnormal enhancement of the bone, paraspinal soft tissues, thecal sac, or epidural region. All fluid collections had resolved. No displacement of the thecal sac was seen.

He completed six weeks of therapy with IV meropenem followed by oral ciprofloxacin and trimethoprim/sulfamethoxazole to complete a total of a six-month treatment course with subsequent full resolution of his symptoms. Repeat MRI at the end of therapy and nine months after the initial presentation demonstrated resolution of the acute bone, epidural, and soft tissue infection/inflammation and stability of chronic discitis changes at L5-S1 (Figure 5).

## Discussion

Our patient developed bacterial discitis with an extremely rare organism, known to cause this infection in the absence of risk factors, and had a very atypical clinical course with complications. Therefore, we aim to emphasize the importance of early detection of these challenging infectious etiologies and highlight the diagnostic utility of the non-invasive microbial cell-free DNA sequencing tests. Early pathogen identification allows for targeted antimicrobial therapy and therefore better outcomes and prognosis.

Discitis is uncommon in the pediatric population, with an incidence of about one to two cases per year in a tertiary pediatric referral center [6]. Its diagnosis can be challenging, leading to delayed diagnosis or unnecessary treatments. The cause may be multifactorial; hypotheses for its etiology range from purely infectious to inflammatory and traumatic [7]. However, most authors attribute etiology to an infectious process [8]. Studies indicate that spinal infections in children begin in the vertebral body near the endplate, in the form of micro-abscesses [9]. Rupture of the focus of infection may then spread the infection from the endplate to the disk through the perforating vascular channels, and its further extension to the paravertebral area, epidural space, and vertebral bodies in proximity [10]. Compression of the spinal cord or nerve root may also follow spinal instability caused by erosive changes in the disk, bone, and posterior elements [11].

The symptoms of discitis include the inability to walk, abdominal pain, chronic back pain, irritability, fever, and local tenderness [12]. The primary site of involvement in children is the L3-L4 and L4-L5 interspaces [3]. In general, three major groups of microorganisms cause spinal infections: bacteria (pyogenic infections), fungi, and (very rarely) parasites [13]. Multiple attempts to determine the causative organisms of pediatric discitis through cultures including blood and/or vertebral aspiration cultures have failed, making the selection of the appropriate antibiotic choices challenging [14]. *Staphylococcus aureus* was found in 80% of the positive cultures [15]. Other less frequently identified organisms include coagulase-negative *Staphylococcus*, α-hemolytic *Streptococcus*, *Streptococcus pneumoniae*, and gram-negative rods such as *Escherichia coli* and *Salmonella *spp. [14]. Another common isolate for spondylodiscitis or discitis in children is *Kingella kingae* [1]. Non-pyogenic bacteria such as *Mycobacterium tuberculosis*, *Brucella* spp., and fungi (i.e., *Aspergillus* spp., *Candida* spp., and *Cryptococcus neoformans*) can cause subacute and chronic discitis [15].

If there is a high clinical suspicion of spinal infection with the possibility of epidural abscess, then MRI of the affected spinal region (cervical, thoracic, or lumbar) with and without intravenous contrast injection is the diagnostic imaging study of choice according to the 2021 American College of Radiology Appropriate Use Criteria [16]. MRI is able to detect early disease whereas X-ray findings lag behind two to eight weeks. MRI has a sensitivity of 100%, specificity of 91.7%, a positive predictive value of 95.2%, and a negative predictive value of 100% [17]. If MRI is non-diagnostic or inconclusive, whole-body 18 F-fluorodeoxyglucose positron emission tomography/computed tomography (FDG-PET/CT) has a sensitivity of 94.8% and a specificity of 91.4%. It is not as sensitive at detecting epidural abscesses and is superior to MRI for identifying multifocal disease.

X-ray evaluation is useful in the identification of alignment abnormalities or when the clinical picture is not specific for discitis or CNS pathology [2]. Because of the low yield, potential morbidity, and need for conscious sedation or general anesthesia in the young child, a biopsy is not routinely recommended for the evaluation of the child with discitis [2]. A biopsy is reserved for patients who do not respond to empiric intravenous anti-staphylococcal antibiotics because an unusual or highly virulent organism may be present [17].

The goals of treatment are eradication of infection and minimization of morbidity. The variable responses to a myriad of treatment protocols in previous studies of discitis in the pediatric population have resulted in continued confusion and discussion as to its underlying etiology [18].

Given the difficulty in finding the underlying infective etiology of discitis in most cases, the newer non-invasive DNA sequencing tests can be useful in timely diagnosis and correct antibiotic choice for better outcomes. In our patient, this test detected *K. aerogenes* as the pathogen and was later confirmed by tissue cultures. Untreated discitis can have serious complications that range from spinal deformities and segmental instabilities to devastating neurologic complications with the spread of the infection to the spinal canal. Spread of the infection to the bone locally can lead to osteomyelitis. Additionally, it can lead to the formation of epidural abscesses, which was the case in our patient.

*K. aerogenes* is a gram-negative, non-motile, encapsulated, rod-shaped bacterium that was formerly known as *Enterobacter aerogenes.* It causes opportunistic infections since it is normally found in the gastrointestinal tract of healthy people. It causes infections of the urinary tract, lung, and bloodstream, and, in addition, it causes infections associated with venous catheter insertion and/or surgical procedures. However, spinal infections caused by *K. aerogenes *are very rare [1], as mentioned earlier in the report.

Despite the early diagnosis, our patient’s course was complicated with epidural abscess likely secondary to emerging resistance to ceftriaxone and later cefepime. The main limitation of the cell-free DNA sequencing tests is the inability to test susceptibility to antibiotics. Broader spectrum antibiotics may be useful when treating serious infections with atypical bacteria detected via cell-free DNA tests to overcome this limitation. However, further induced antibiotic resistance needs to be considered and antibiotic stewardship should be employed.

## Conclusions

Discitis is an uncommon disease in the pediatric population. Its timely diagnosis is very important and simultaneously very difficult since the clinical presentation can be non-specific and the laboratory data may not be helpful in diagnosing the disease. Therefore, diagnosis is largely dependent on high clinical suspicion paired with MRI findings. Since only 50% of the cultures from the biopsy specimen, intraspinal fluid, or aspiration fluid reveal the etiology making the treatment even more challenging, newer DNA sequencing tests can be employed to aid the diagnosis and guide correct antibiotic treatment. Broader spectrum antibiotics may be necessary if the symptoms persist in the absence of cultures and susceptibilities. Untreated discitis is associated with serious morbidities, and therefore a timely diagnosis is paramount.
